# MicroRNAs; easy and potent targets in optimizing therapeutic methods in reparative angiogenesis

**DOI:** 10.1111/jcmm.12669

**Published:** 2015-09-28

**Authors:** Fatemeh Pourrajab, Abbas Vakili Zarch, Seyedhossein Hekmatimoghaddam, Mohamad Reza Zare‐Khormizi

**Affiliations:** ^1^ School of MedicineShahid Sadoughi University of Medical SciencesYazdIran; ^2^ Department of Clinical Biochemistry and Molecular BiologySchool of MedicineShahid Sadoughi University of Medical SciencesYazdIran; ^3^ Department of Laboratory SciencesSchool of ParamedicineShahid Sadoughi University of Medical SciencesYazdIran

**Keywords:** microRNAs, treatment strategies, angiogenesis

## Abstract

The age‐related senescence of adult tissues is associated with the decreased level of angiogenic capability and with the development of a degenerative disease such as atherosclerosis which thereafter result in the deteriorating function of multiple systems. Findings indicate that tissue senescence not only diminishes repair processes but also promotes atherogenesis, serving as a double‐edged sword in the development and prognosis of ischaemia‐associated diseases. Evidence evokes microRNAs (miRNAs) as molecular switchers that underlie cellular events in different tissues. Here, miRNAs would promote new potential targets for optimizing therapeutic methods in blood flow recovery to the ischaemic area. Effectively beginning an ischaemia therapy, a more characteristic of miRNA changes in adult tissues is prerequisite and in the forefront. It may also be a preliminary phase in treatment strategies by stem cell‐based therapy.

## Differentially expressed miRNAs act as switchers or fine‐tuners

Atherosclerosis is the primary cause of cardiovascular disease (CVD), carotid artery disease and peripheral vascular disease 1.

On the vascular site, coronary artery disease (CAD) and atherosclerosis share a major pattern of inflammatory component and also an interesting pattern of circulating microRNAs (miRNAs). The most shared miRNAs are endothelial cell (EC) derived and include miR‐126/17–92/15/34 clusters as representing highly circulating miRNAs 2, 3, 4, 5, 6.

All the underlying mechanisms involved in the pathophysiology of atherosclerosis, have been defined to be modulated by miRNAs, directly or indirectly. The short, non‐coding RNAs regulate gene expression through translational repression or degradation of target mRNA. As master regulators of many biological processes and because of their ability to target a network of mRNAs involved in angiogenesis, miRNAs are assumed to control ischaemia/hypoxia conditions 7, 8.

The targets of miRNAs are usually transcription factors which are part of a feedforward loop. It has been demonstrated that there is a mutually negative/positive feedback loop between miRNA and its target 9, 10. Thus, small changes in a miRNA level can have large physiological effects and can thereby confer robustness to biological processes, like a cell fate switcher. Especially when a target mRNA is in low levels and the repression of a miRNA is in the strongest level. Then, miRNAs can act as switchers or fine‐tuners depending on target levels 11, 12. Moreover, epigenetic machinery such as DNA/histone methylation or acetylation is also shown to be regulated by miRNAs, directly or indirectly. The epigenetic machinery can induce global changes in gene expression 13, 14. Despite many targets for a specific miRNA, target reconstitution experiments have shown dominant roles of a particular target in a specific tissue, as a major player in the effect of miRNA 7, 15, 16.

Comprehensive evaluation of miRNA targets has revealed that these biomarkers not only control cell cycle traverse and proliferation, but also DNA repair and apoptosis processes 17.

## Ischaemia/hypoxia‐associated miRNA signatures in atherosclerosis

Ageing is associated with senescence and is also related to the increased levels of oxidative/nitrosative stress. Atherosclerosis is in turn associated with ageing and its related pathways in humans. Endothelial senescence is a major risk factor for atherosclerosis and CVD. Oxidative/nitrosative stress induces EC senescence which causes vascular dysfunction, endothelial expression of adhesion molecules and leucocyte adherence to the arterial walls in humans 16, 18.

Accordingly, a set of miRNAs are involved in the various stages of atherosclerosis, increasingly up‐ or down‐regulated during the disorder progression.

In this regard, some particular miRNAs including let‐7f, miR‐15, ‐34, ‐146, ‐200, ‐217, ‐222 (generally up‐regulated), or miR‐17–92, ‐21, ‐133, ‐126, ‐214 (generally down‐regulated), have been associated with angiogenesis disorders in humans, especially by stress‐induced premature senescence 12, 13, 17, 19, 20.

All of them are found to be differentially regulated under ischaemic/hypoxic conditions. For example, a blood miRNA profiling of young ischaemic stroke patients (18–49 years) has revealed high expression of let‐7, ‐21, ‐222, ‐181 *versus* poorly expressed miR‐126, ‐186 20. Or, miRNA profiling of blood samples from patients with atherosclerosis has exhibited the differential expression of specific signature let‐7, miR‐15/16, 23–27, ‐126 7. Studies have also verified a similar pattern of high‐ and low‐expressed miRNAs in peripheral endothelial progenitor cells (EPCs) from patients with atherosclerotic conditions 7, 25.

According to studies, let‐7, miR‐126, ‐130, ‐150, ‐17–92, ‐217 and ‐222 regulate endothelial function and angiogenesis, whereas miR‐15, ‐21, ‐23–27, ‐34, ‐146, ‐181 are more involved in vascular remodelling and immune responses 4, 13, 20, 21, 22, 23. For instance, induced senescence in human ECs has been addressed to reduce expression levels of proliferation‐stimulating/apoptosis‐suppressing miR‐17–92, ‐21 and ‐214, in contrast to tumour suppressor p16 and miR‐222 family for suppression of endothelial nitric oxide synthase (eNOS) 23. Down‐regulation of miR‐126 and ‐130a, in particular, impairs EC proliferation, migration and angiogenesis. Anti‐inflammatory miR‐126 and ‐133a targets Spred‐1/caveolin‐1, which respectively represses Ras/extracellular signal‐regulated kinase (ERK)/VEGF and PI3K/Akt/eNOS signalling pathways 7, 25.

A distinct set of miRNAs including let‐7, miR‐17–92, ‐27, ‐130 and ‐222 has been introduced with compensatory effects on arteriogenesis to regulate vascular development 8, 24.

Furthermore, mito‐miR‐34a, ‐146a and ‐181 are known as pro‐inflammatory markers of senescence status and to be involved in vascular remodelling. The mito‐miR‐34a, ‐146a, and ‐181a are important metabolic and cell death regulators in CVD. A number of mito‐proteins which play large roles in energy metabolism, mitochondrial transport and apoptosis are targets of mito‐miRNAs. Bcl‐2 family is one of them, critically involved in maintaining mitochondrial integrity 26, 27, 28.

Specifically, miRNA family miR‐15, ‐34 and let‐7 *versus* miR‐17–92 and ‐106 are found to be major players in cell senescence and arresting cell cycle by targeting genes that are essential for cell cycle traverse 5, 16, 17.

Notably, up‐regulation of miR‐15 family has been observed in both hypoxic and ischaemic condition. From the aforementioned data, therapeutic benefit is achieved by miR‐15/16 inhibition and by the transplantation of progenitor cells *ex vivo*‐manipulated with anti‐miR‐15/16 agents, which thereby influence the post‐ischaemic recovery of mice with ischaemia condition 29.

Identification of key miRNA signatures and their targets involved in the development and progression of atherosclerosis is important as this sheds light into the underlying mechanisms of ischaemic diseases and related complications.

### Potent genes targeted by senescence‐associated miRNAs in atherosclerotic pathological conditions

Endothelial senescence is the major hallmark of CVDs such as atherosclerosis 2, 31. The well‐established tumour suppressors miR‐15 and ‐34 are potent triggers of EC senescence; through repressing the mRNAs E2F, c‐Myc, Sirtuin 1 (SIRT1), Cdk4, Cdk6, Bcl‐2, hepatocyte growth factor receptor (Met), and cyclins D1 and E2 14, 33. The senescence‐associated transcription of miR‐34 by p53 and miR‐29/miR‐30 transcription by RB are the best‐documented examples thus far 34.

The miR‐15 and ‐34 families exert tumour suppressor activity in multiple adult tissues 4, 5, 28, 33. In clinical studies on patients with CVD, the anti‐atherosclerotic and vasodilatation effects of statins have been addressed to induce the bioactivity of NOS and Bcl‐2 in endothelium. A recent study has revealed the *in vivo* anti‐senescent properties of statins. Statins enhance the expression of SIRT1 and repress the expression of particular miRNA families, including miR‐15 and ‐34, in EPCs from patients with CVD 4, 30.

These miRNAs are linked to increased apoptosis, reduced level of eNOS activity and inflammation and are thereby associated with the senescent phenotype in human ECs 2, 23.

The elevated levels of miR‐15, ‐34, ‐217 have been linked to CVD in humans for targeting SIRT1 which is a negative regulator of reactive oxygen species (ROS) production and up‐regulation of apoptotic proteins. Down‐regulation of SIRT1 and inhibition of cell proliferation are two major causes that ultimately contribute to atherosclerosis 4, 13, 16, 32. SIRT1 has recently been reported to be a novel modulator of vascular EC homoeostasis, and has been shown to exert anti‐atherosclerotic effects against endothelial dysfunction by preventing stress‐induced senescence *in vivo*/vitro 2, 4, 13, 31, 35.

Reduced level of miR‐15 family allows accumulation of Bcl‐2 which causes increased resistance of senescent cells to apoptosis 5. The apoptosis effect of the miR‐15 family has been addressed to down‐regulate SIRT1, cell cycle regulators and Bcl‐2 5, 32.

On the other side, miR‐34 by activating the p53/p21 pathway, acts as a potent trigger of senescence in human cells 34.

The stimulated expression of miR‐15, ‐34 and ‐217 has been strongly associated with hypoxic/ischaemic‐induced stress conditions 29, 31, 36. It has been determined that these specific miRNAs are able to affect mitochondrial integrity in the cardiovascular (CV) system 4, 13, 37, 38. In detail, these miRNAs are highly expressed in ECs during induced cell senescence for inhibiting directly deacetylase SIRT1, thereby to increase acetylation of SIRT1 target genes and to suppress angiogenesis 13, 32, 33, 34, 35. Accordingly, increased acetylation of SIRT1 target genes such as p53 and FoxO, promotes miR‐15, ‐34, ‐217 expression for instance, there is a positive feedback loop between p53 and miR‐34, to accelerate cellular senescence 10, 31, 34, 39. The other set of potent targets of miR‐34a include Sp1, Nrf2, SIRT1 and Mgst1 whose expression is inversely related to the up‐regulation of miRNA 39. Interestingly, miR‐34a not only represses genes modulating oxidative stresses such as SIRT1 and Mgst1, but also their upstream activators, like as Sp1 and Nrf2 transcription factors 10, 33, 39. Then as a double dampening regulation, up‐regulated miR‐15, ‐34, ‐217 constitute an inescapable repression loop of the vital oxidative defence genes, by targeting not only them but also their upstream transcription factors controlling their activation 10, 39.

SIRT1 and Mgst1 are two major components of the signalling network to counteract the damaging effects of stress 4, 39. SIRT1 and Mgst1 changes are precipitating in ageing, insulin resistance and detoxification process of ROS/reactive nitrogen species (RNS) 33.

Targeting SIRT1 can thereby disrupt the deacetylation and regulation of a broad range of transcription factors including; p53, FoxO, the nuclear receptors liver X receptor (LXR), oestrogen receptor α, and androgen receptor, the hypoxia‐inducible factor α, the coregulator Peroxisome proliferator‐activated receptor gamma, coactivator 1 alpha (PGC1α), and the DNA repair machinery 35, 37.

As well, there is a broader role for SIRT1 in epigenetic regulation for example SIRT1 can modulate chromatin remodelling by direct deacetylation of histones and recruitment of nuclear enzymes for histone and DNA methylation 32, 35, 37.

Of importance, *in vivo* CpG hypermethylation of miR‐34a promoter has been found in primary malignancies, consistent with low transcription of miR‐34a. These findings indicate a close negative regulatory loop in the SIRT1–p53 pathway capable of regulating miR‐34a expression 33, 34.

However, comparing EPCs from young with old healthy individuals has revealed a significant down‐regulation of miR‐24, ‐130a, ‐155 and ‐221/222 in healthy old individuals. The targets of these miRNAs are predicted to be PI3K, c‐Kit and H2AX which are needed to be elevated with advancing age to protect and support cells against ageing processes 12. The miR‐221/222 family is, in fact, highly expressed in vascular system and their roles in vascular physiology have been extensively studied 38 (Table 1).

**Table 1:**
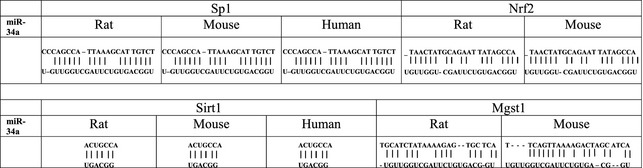
The conserved target sites on 3‐UTRs of Sp1/Sirt1 (rat, mouse and human) and Nrf2/Mgst1 (rat and mouse), transcripts targeted by miR‐34a, across species 39

Decreased level of miR‐221s in healthy old individuals, is sufficient to increase the expression of their predicted targets; PI3K and c‐Kit 2, 12, 38. In contrast, higher levels of c‐Kit protein are observed in healthy old individuals. Overexpression of miR‐221/222 in ECs targets mainly c‐Kit which is essential for stemness proper‐ties of c‐Kit^+^ ECs and their ability to form new capillaries 11. Intriguingly, PI3K is downstream of the tyrosine kinase receptor c‐Kit. Therefore, up‐regulation of PI3K potentiates even more c‐Kit signalling in PCs with advancing age 12.

In contrast to ECs, the miR‐221 family has been found to induce pro‐proliferative, pro‐migration and anti‐apoptotic effects in vascular smooth muscle cells (VSMCs) 38. The opposite effects observed for the miR‐221 family in VSMCs have been related to different expression profiles of their target genes in two cell types, p27, p57 (anti‐proliferative) and c‐Kit (pro‐proliferative) 2, 40. The p27/p57 *versus* c‐Kit is highly expressed in VSMCs, while c‐Kit *versus* p27/p57 is highly expressed in ECs. Thus, different availabilities of these target gene mRNAs in VSMCs and ECs are related to the different cellular effects of miR‐221/222 in these two cell types 38.

### Cell cycle withdrawal by ischaemia‐induced miR‐15 sequence‐shared miRNAs

Consistently, the miR‐15 family has been reported to be increased in PCs and serum of patients with ischaemic conditions, which is associated with the impairment of human circulating pro‐angiogenic function of PCs 29 The family members overexpressed by muscles in response to ischaemic condition act to negatively regulate bone marrow‐derived PC response to chemotactic stimuli fibroblast growth factor (FGF), VEGF‐A and stromal cell‐derived factor‐1α (SDF‐1α) 29.

The major targets of the miR‐15 family are predicted to be regulators of cell cycle in mitotosis, chromosome condensation and cellular response to DNA damage 5, 32, 41.

The family consists of conserved miRNAs (miR‐15, ‐16, ‐195, ‐497), which is expressed in most adult tissues, particularly in vascular system. The members share a common seed region, with varying degrees of sequence homology in the non‐seed region of the mature miRNA 5. The family shares a portion of their seed sequence with miR‐503 and miR‐424 29. Likewise, the sequence‐shared miRNAs have been reported to be up‐regulated in patients with ischaemia condition, particularly in atherosclerosis 44.

It has been demonstrated that miR‐16 and miR‐424 inhibit *in vitro* angiogenesis by suppressing the expression of VEGF‐A and FGF receptor‐1 (FGF‐R1) 29. Data indicate that the miR‐15 family act as negative regulators of angiogenesis through (*i*) inhibiting the expression of VEGF‐A and transcription factors E2F1/3 and (*ii*) through regulatory mechanisms governing cell cycle withdrawal by the activation of p53 5, 32, 41, 42. Particularly, high levels of miR‐503 observed in the plasma samples from ischaemic patients are attributed to impairing the normal function of ECs 45.

Up‐regulated miR‐503 disturbs molecular mechanisms necessary for cell cycle progression and EC function in reparative angiogenesis 44. For example, a number of highly conserved cell cycle genes, including checkpoint kinase Chk (Cdc25 phosphatase), as well as, Cdc2a and Spag5 are miR‐15 targets 5, 42. Chk is one of the most important effectors inactivated by ATM kinase. Upon DNA damage, ATM activates p53 and inactivates Chk (Cdc25) respectively. Thereby, the functional inhibition of Cdc25 prevents the activity of CDKs (Cdk4/6) involved in G1/S transition, and progression through S and G2/M(Cdk2) phases. Inhibition of Cds25 precipitates cell cycle at G1/S or at G2/M arrest by triggering checkpoints 33, 41, 43. At the same time, ATM‐mediated activation of p53 is required for the induction of DNA damage response. With a prominent role in mediating DNA damage response in oxidative stress and telomere erosion, however, over‐activation of major tumour suppressors p53 and Rb, leads cell progression towards senescence 42, 43, assuming miR‐15 is induced to function downstream of the p53/Rb pathway 5, 32, 41.

**Figure 1:**
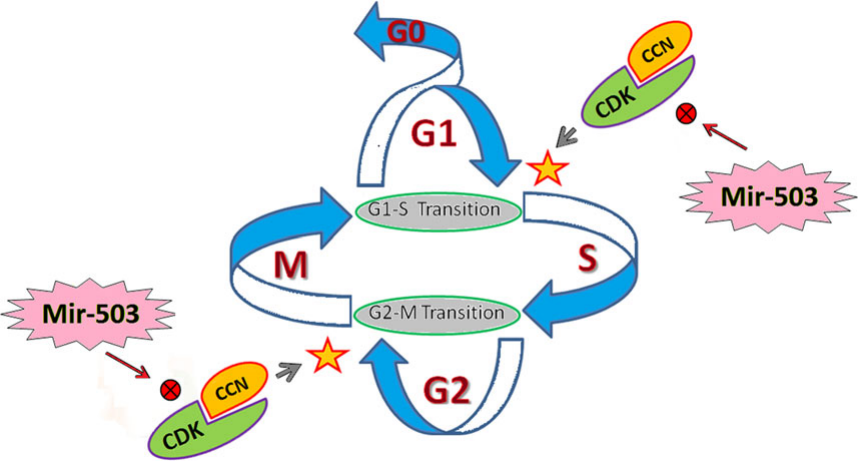
The putative targets of the sequence‐share miRNAs; miR‐15 family, miR‐424 and miR‐503, are major players in cell cycle. In endothelial cells, under stress conditions such as ischaemia, the sequence‐share miRNAs are up‐regulated which target cell cycle regulators CCN‐D, CCN‐E and Cdc25a. The G1/S and G2/M transitions are the most important check points in cell cycle arrested by up‐regulated miR‐503/15, through modulation of Cdc25a, CCN‐E and CCN‐D. CCN‐D/Cdk4/6 integration results in the early progression of G1, while CCN‐E activates Cdk2 in the late G1, leading to the G1/S transition, in particular. CCN‐A/Cdk2 complexes assure S phase progression, and CCN‐B/Cdk1 integrate completes mitosis (M). The phosphatase activity of Cdc25a is critical for timely activation of Cdk1/2. Cdks Cyclin‐dependent kinases; CCNs: cyclins.

Entry into each phase of the cell‐cycle is regulated by the subsequent expression of cyclin‐dependent serine/threonine kinases (Cdks), their binding partners cyclins (CCNs), and associated regulatory proteins, all of which would be influenced by the miR‐15 family and the sequence‐shared miRNAs 5, 32, 34, 42. The cell cycle progression is tightly regulated, by modulating the activity of Cdc25a, CCN‐E and CCN‐D. CCN‐D activates Cdk4/6 in early G1 for progression in this phase. CCN‐E is needed to activate Cdk2 at the late stage of G1, in particular, for G1/S transition and passage into the S phase of cell cycle 41, 46. Thereafter, CCN‐A/Cdk2 complex assures S phase progression, and CCN‐B/Cdk1 integration completes mitosis. However, among Cdks and CCNs, the phosphatase activity of the cell division cycle 25 family (Cdc25) is critical for the timely activation of regulators Cdk1 and Cdk2. Thus, Cdc25a mainly regulate G1‐S and G2‐M transition. The cell cycle progression is tightly regulated, through modulation the activity of Cdc25a, CCN‐E, CCN‐D 41, 42, 46, 47, 48. Accordingly, some sequence‐shared miRNAs such as miR‐15s, miR‐503 and miR‐424 potentially target cell cycle regulators; cyclin CCN‐E and phosphatase Cdc25 in ECs when these cells confront stress conditions such as high glucose levels or low levels of growth factors (Fig. 1) 5, 29, 42, 45.

Of significance, *ex vivo* experiments exhibit that inhibition of miR‐15/16 empowers PCs and increases their therapeutic potential when they are transplanted in an animal of ischaemia model 29. Moreover, anti‐miR‐503 delivery to the ischaemic mice has reported to correct impairment of angiogenesis and cause blood flow recovery 45.

## miRNA signatures related to pathological inflammatory conditions of atherosclerosis

Indeed several miRNAs have been emerging to be involved in inflammation, because of their ability to modulate the expression of important pro‐inflammatory molecules such as the nuclear factor‐kappa B (NF‐KB) (miR‐9), TLR‐4 (let‐7), vascular cell adhesion molecule‐1 (VCAM‐1) (miR‐126), Monocyte Chemoattractant Protein (MCP)/VCAM‐1 (miR‐21), Interleukin‐1 receptor‐associated kinase 1 (IRAK‐1) (miR‐146) and several others. Increasing levels of these specific miRNAs are commonly observed in patients with age‐related diseases 11, 28.

The majority of miRNAs detected in the blood of patients with ischaemic conditions, belongs to let‐7, 15, 23, 34, 181, 221 families and is of vascular origin. Whereas, differentially expressed miRNAs in inflammatory conditions include miR‐17–92, ‐21, ‐126, ‐146a, ‐155, ‐150, ‐221s 2, 3, 20, 26, 39. They have critical roles in controlling function and phenotype of VSMCs and ECs in atherosclerosis development 50, 51, 52.

For example, miR‐21, ‐92, ‐126, ‐146a, ‐155 and ‐221s are up‐regulated in human atherosclerotic plaques and have been determined as key modulators in atherogenic events 11, 23, 28, 49.

In CAD, an interesting pattern of miRNAs is present, while most of them are EC derived. For example, highly circulating miRNAs such as miR‐17–92 would decrease in the blood of patients with CAD 3.

Likewise, miR‐126 has been observed to be reduced significantly in the blood samples from young patients with stroke, as well as, CAD 11, 20. The miR‐126 control vascular inflammation by regulating the expression of adhesion molecules requiring for leucocyte adherence to ECs, such as VCAM‐1 2.

Notably, the endothelial expression of adhesion molecules would be increased in the arterial walls of humans during ageing process, 16, 18.

The EC‐expressed let‐7, miR‐17–92, ‐34, ‐133, ‐146, ‐181 and ‐221 clusters have been defined as mito‐miRNAs or senescence‐associated miRNAs 28. Between them, miR‐126, ‐17–92 and ‐221 are most known to control the growth of new blood vessels 23.

The miR‐17–92, ‐126, ‐130a and ‐214 signatures *versus* miR‐222, ‐217, ‐34a, ‐15s play the anti‐senescent and angiogenic role in vascular system 8, 15.

The anti‐inflammatory miR‐126 is also a negative regulator of endothelial senescence, and is reduced in senescent ECs, while pro‐inflammatory miR‐146, ‐221 are increased 23, 26.

miR‐126 is also the most abundant miRNA in apoptotic bodies which induces CXCR4 axis for increasing CXCL12 expression. The CXCL12/CXCR4 axis is highly implicated in PC incorporation in atherosclerotic plaque for conferring a less inflammatory response and more stable plaque phenotype 11.

Vascular inflammation includes participation of three factors MCP‐1, VCAM‐1 and matrix metalloproteinase (MMPs), whereby inflammation is inhibited by miR‐126 by targeting VCAM‐1 translation.

Accordingly, tumour necrosis factor (TNF)‐α is an inducer of transcription factor Ets‐1 which in turn mediates both the expression of pro‐inflammatory genes MCP‐1 and VCAM‐1 and up‐regulation of miR‐126. The net effect of vascular inflammation is a balance between Ets‐1‐induced pro‐inflammatory and Ets‐1‐induced anti‐inflammatory effects 2, 23. Ets‐1 as an inducer of miR‐126, is down‐regulated by miR‐155 and ‐221/222 2, while miR‐21 targets pro‐inflammatory mediators MCP‐1/VCAM‐1 2, 31. Moreover, miR‐221/222 targets eNOS and impairs NO bioavailability which is a causative role in endothelial dysfunction and a hallmark of CAD in patients with atherosclerosis 23, 38.

Furthermore, in obese mouse model, the miR‐221 family is overexpressed and is responsible for inducing inflammatory cytokines besides increasing amounts of miR‐27 38.

The miR‐221 is highly up‐regulated in dendritic cells (DCs) upon differentiation from human monocytes, while miR‐21, ‐25, ‐17–92 are highly expressed in human immature circulating monocytes. The miR‐221, ‐155 and ‐146 are associated with maturation of monocytes/DCs 40. The last two miRNAs are up‐regulated in innate and adaptive inflammatory responses upon activation of immune systems. Elevated levels of miR‐155 are characteristic of pro‐inflammatory macrophages and atherosclerotic lesions in hypercholesterolaemia patients 49, 54.

The mir‐146a is among inflammatory miRNAs primarily involved in ageing 28. Circulating angiogenic cells from patients with chronic heart failure who presenting distinguishing features of senescence, remarkably overexpress miR‐146a. Moreover, significant correlations have been detected between miR‐146a expression with telomere length and telomerase activity, as well as, inflammatory statues 26, 27, 28, 31. There is also a relation between highly expressed Noxs and miR‐155 in leucocytes of patients with hypercholesterolaemia 49, 54 and up‐regulation of oxLDL uptake and ROS production 49. The Nox isoforms are the major source of ROS production within the CV system and are related to reperfusion injuries 27, 38, 53.

Nox isoform presented in VSMCs is the potential target for miR‐146a, while the isoform expressed in ECs and leucocytes remains unaffected 27, 53, 54. Increased expression of Nox in ECs leads consequently to oxidative/nitrosative stress in the heart 54. However, miR‐146a and 181s which induce expression of inflammatory mediators IRAK‐1 and TNF receptor‐associated factor‐6 are also up‐regulated in ageing cells 28.

## Molecular signatures associated with PC homing

Stromal cell‐derived factor‐1α is a powerful chemoattractant for PC requirement, essential for mobilization from the bone marrow, trafficking in blood and homing in ischaemic tissues 60.

Hypoxia‐inducible factor‐1α (HIF‐1α) induces SDF‐1α expression to increase adhesion, migration and recruitment of circulating CXCR4‏ PCs to hypoxic sites. Inhibition of SDF‐1α in ischaemic tissues or blockage of CXCR4 on circulating PCs, prevents PC homing in sites of injury 61, 62, 63. However, a subset of miRNAs regulated by HIF‐1α are important regulators of endothelial function, especially angiogenesis 69.

A recent *in vivo* cell therapy‐based study has suggested that the administration of adenosine‐treated PCs would represent a more promising option to regenerate the heart after myocardial infarction 62.

According to the record, adenosine triggers angiogenesis in ischaemic heart by stimulating the PC expression of CXCR4 and A2B receptors *versus* miR‐150. The miR‐150 reduces PCs homing in inflammatory niches by targeting SDF‐1α/CXCR4 axis.

CXCR4 is a direct target of miR‐150, ‐146a, ‐133 and ‐1 in PCs 57, 61, 64, whereas miR‐23 (miR‐23, ‐24, ‐27) and 221 (miR‐221, ‐222, ‐223) targets SDF‐1α 63, 65, 66. Thereby, up‐regulation of these miRNAs *versus* miR‐126 in ischaemia respectively represses PC requirement, directional migration and retention of PCs within injured sites 65, 66. The SDF‐1α/CXCR4 axis, in particular SDF‐1α, is essential for requiring c‐Kit^+^ PCs into the circulation, for homing into the site of injury and for stem cell participation in the regeneration of injured tissues 65, 67.

But, overexpression of miR‐126 facilitates vascular regeneration accordingly by increasing the number of circulating c‐Kit^+^ PCs. Intriguingly, miR‐126 attenuates the expression of CXCR4 on c‐Kit^+^ cells situated in the bone marrow while increasing the expression of SDF‐1α in ischaemic tissues, triggering mobilization of c‐Kit^+^ cells from the bone marrow and favouring their homing into the injured site 59, 68.

Interestingly, apoptotic bodies enriched in miR‐126 mediate an auto‐regulatory positive feedback loop to trigger SDF‐1α/CXCR4 axis 61, 62, 64.

Considerably, adenosine‐treatment of PCs represents a more efficient cell‐based therapy since triggering SDF‐1α/CXCR4 axis 62. Whereas, overexpressed miR‐150, ‐146a, ‐221s and ‐23s under ischaemic conditions, would suppress SDF‐1α/CXCR4 axis in c‐Kit^+^ PCs 56, 59, 61, 65, 66. On the other hand, miR‐126 overexpression stimulates SDF‐1α/CXCR4 axis, directionally to promote incorporation of c‐Kit^+^ PCs into inflamed sites such as plaques to limit atherosclerosis and conferring plaque stability 59.

## Some miRNA key mechanisms in reparative angiogenesis

Experiments raise the possibility that indirect mechanisms by promoting direct pro‐angiogenic treatments could contribute to better *in vivo* effects 56, 57, 58, 59.

Data indicate that administration of angiogenic circulating PCs in combination with miRNAs would stimulate neovascularization to improve ischaemia condition 4, 26, 55. In this regard, in the mouse model of myocardial infarction, down‐regulation of miR‐199a and up‐regulation of miR‐17–92 de‐represses the expression of both SIRT1 and HIF‐1α which causes a preconditioning state for recovery of the heart after ischaemia 74.

Considering that adult ECs would be at the lowest level of their proliferation capacity, in patients with chronic ischaemia conditions, the quiescence state is induced by miR‐15, ‐34a, ‐217, ‐424 and ‐503 overexpression 4, 5, 13, 28, 32, 33, 71. Notable in animal models and in patients with chronic ischaemia condition, increased levels of miR‐15s and ‐503 have been observed in plasma and circulating c‐Kit^+^ cells 29.

Consistent data indicate that the quiescent state of EPCs is rapidly reversed in response to growth factors; VEGF and IGF‐1, the robust switchers of quiescent c‐Kit^+^ PCs towards the activation state 71, 72, 73. IGF‐1 protects c‐Kit^+^ stem cells against ROS‐induced mitochondrial dysfunction and inhibits mitochondrial‐induced apoptosis, through PI3K/Akt pathway 72.

Data also define the PI3K/Akt pathway as a key player in cell survival under oxidative/nitrosative stress. The pathway is regulated directly by the miR‐17–92 family. Oxidative stress correlates with increased expression of Phosphatase and tensin homolog (PTEN) and inhibition of Akt signalling. PTEN is predicted to be a direct target of miR‐17–92 21. Highly expressed miR‐17–92 in ECs from young healthy individuals have been associated with being free of disease 50. In heart samples obtained from patients with chronic‐ischaemia cardiomyopathy, remarkable down‐regulation of miR‐17–92 has been reported 70. The cluster is the most down‐regulated miRNAs in organism models of ageing and in human senescence, indicating that these processes are intricately interwoven 17, 28, 75, 76. The upstream transcriptional start site of the cluster is highly conserved across vertebrates, contains an extensive CpG island, a core putative promoter regulated by epigenetic modifications 50, 69. Notably, forced‐expression of miR‐92a alone, in ECs was found to block angiogenesis in ischaemia mouse model which has been corresponded to targeting pro‐angiogenic integrin subunit alpha5 24.

Increased expression of miR‐17–92 cluster is associated with cell growth and proliferation, while its decreased expression is associated with cell senescence 17, 50. In contrast to up‐regulating miR‐15, ‐34, ‐181 families, the miR‐17–92 cluster is down‐regulating with advancing age and in PBMCs from old individuals 12, 75, 76, 77. Furthermore, the members of miR‐17–92 cluster make up a negative feedback loop to control endothelial inflammation through negatively modulating adhesion and inflammatory responses in leucocytes (inhibiting p‐selectin glycoprotein ligand (SELPG) and ICAM‐1) 2, 28. Additionally, miRNA profiling of circulating human PCs, which proposed to be used in CV regenerative process, exhibited higher expression of miR‐126 and the miR‐17–92 family 78.

In addition, reduced levels of miR‐126, ‐130a and ‐21 in EPCs obtained from patients with ischaemic conditions specifically impair endothelial function 25, 79. The main molecular mechanisms induced in EPCs by miR‐126 and ‐130a has been addressed to up‐regulate ERK/VEGF and Akt pathways 25. They promote angiogenesis by repressing directly important inhibitors of angiogenesis including Spred‐1(miR‐126) and HOXA5 (miR‐130a) 80. Spred‐1 suppresses ERK/VEGF pathway, and HOXA5 acts through several mechanisms such as down‐regulation of pro‐angiogenic factors, such as VEGFR2, Ephrin A1 and HIF‐1α, and up‐regulation of anti‐angiogenic molecule thrombospondin‐2 81.

During hypoxic conditions and *via* HIF‐1α pathway, massive expression of miR‐24 occurs in ECs, which directly targets the endothelium‐enriched transcription factor GATA2 (a member of transcription factors family which bind to the DNA sequence "GATA") and the p21‐activated kinases 4 (PAK4), both of which have established roles in vascular biology 69, 82. Notably, local inhibition of massive‐expressed miR‐24 improves reparative angiogenesis and left ventricle remodelling after MI. One of miR‐24 targets in the heart is eNOS 83.

The PAK4 inactivates the pro‐apoptotic factor BAD (the Bcl‐2‐associated death promoter protein), while GATA2 is the putative inducer of SIRT1. Thereby, miR‐24 by silencing simultaneously both GATA2 and PAK4, abrogates strongly cell survival and putatively induces apoptosis. In fact in ECs, the signalling molecules PAK4 and GATA2 are the main members of pro‐angiogenic pathway, whereby angiogenesis is suppressed by miR‐24 overexpression 83.

**Figure 2:**
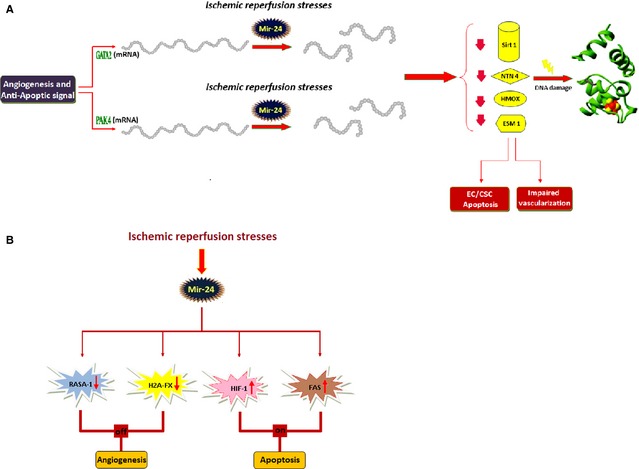
The local inhibition of miR‐24 in ECs would turn off apoptosis signalling pathway and improves reparative angiogenesis in post‐ischaemic conditions. The mechanism used by miR‐24 to switch between angiogenesis and apoptosis in ECs. (**A**) The pro‐apoptotic and anti‐angiogenic effects of miR‐24 in ECs, are mediated by repressing downstream mediators: PAK4, GATA2, heme‐oxygenase‐1 (HMOX1), SIRT1. GATA2 induces the expression of SIRT1 and HMOX1. (**B**) A special miRNA can regulate the multiple genes and the major signalling pathway of a cell. The signal transduction factor RASA1; the chromatin remodelling factor H2AX and HIF‐1α pathway are the other major targets of miR‐24 in ECs.

GATA2 is also a putative inducer of heme‐oxygenase‐1 (HMOX1), a vasoprotective and anti‐apoptotic enzyme. MiR‐24‐mediated repression of HMOX1 results in ROS stress which subsequently predisposes cells to DNA damage and senescence (Fig. 2A) 84.

The signal transduction factor RASA1 (a component of tyrosine kinase receptor pathway) and H2AX (a chromatin remodelling factor) 85, both are direct targets of miR‐24 in ECs 69.

In healthy old individuals, the expression of miR‐24 and ‐155 is normally down‐regulated which co‐ordinately modulate H2AX expression, to overcome the additional oxidative stress and DNA damage that occurs with ageing 12, 33.

Interestingly, administration of miR‐21 and miR‐24 would resemble ischaemic pre‐conditioning to reduce apoptotic region and induce the expression of eNOS, heat‐shock transcription factor‐1, and heat‐shock‐protein‐70, in the animal model of MI 74.

In summary, the major pathways in ECs abrogated by miR‐24 are related to tyrosine kinase receptors or chromatin remodelling factors and their major roles in angioprotection (Fig. 2B).

However, miR‐24 can result in anti‐apoptotic effects in myocardium *versus* ECs, as differential gene expression networks exist between two cell types 69, 86.

In vascular ageing, some specific miRNAs (including miR‐15, ‐21, ‐23, ‐34, ‐29, ‐181/182 and ‐199), show aberrant expression. Among them, miR‐23 (miR‐23, ‐24, ‐27) and miR‐15 (miR‐15, ‐16, ‐195, ‐497) families are generally overexpressed under hypoxia by HIF‐1α pathway 20, 29, 50, 70.

## Angiogenesis and miRNA‐related changes

Angiogenesis is a complex process that depends on the balance of pro‐ and anti‐angiogenic factors that influence the quiescence or proliferative state of the endothelium in a wide variety of disease states. Evidence show that miRNAs are differentially expressed in activated ECs, where they suppress proliferation, or permit activation of signals leading to neovascularization 15, 50, 86.

Therapeutic stimulation of the angiogenesis process represents a novel strategy to support post‐ischaemic BF recovery, wound closure and tissue regeneration in ischaemic complications. Ischaemic complications represent the leading cause of morbidity and mortality in diabetic patients. For example, peripheral arterial disease (PAD) in diabetes mellitus patients characterized by pain at rest, can accordingly lead to critical limb ischaemia, a life‐threatening condition which results in ulcer and gangrene in tissues and their loss 7, 29, 45. On the other hand, ischaemic heart disease (IHD) which triggers dysfunction and the death of cardiomyocytes, becomes the commonest cause of death throughout the world, when a large number of patients are not qualified for the conventional revascularization techniques of balloon angioplasty and stenting, or coronary artery bypass grafting 80, 83.

It is now well‐established that EC dysfunction contributes to vessel wall changes and vascular diseases. The pathological change in the vessel wall during atherosclerosis is the underlying mechanism of ischaemic diseases, especially in IHD and stroke. In addition, impaired neovascularization has been associated with EC dysfunction 15, 80, 83.

In this regard, dramatic changes in miRNA levels could theoretically lead to adverse side effects, for example, overexpression of miR‐133 and/or miR‐132 has been reported to be associated with retinal degeneration. Moreover, anti‐angiogenic agents that target the VEGFR and bFGF pathways have shown considerable clinical benefits in patients with solid tumours or retinal diseases associated with pathological neovascularization 71, 95.

Tumour‐associated angiogenic miRNAs can up‐regulate the endothelium to facilitate pathological angiogenesis. Accordingly, anti‐angiogenic miRNAs that target the VEGFR pathway would exhibit considerable clinical benefit in patients with pathological neovascularization especially in those with tumours or with retinal diseases. Using a single intraocular injection of anti‐miR‐132 and monitoring vascular growth post‐injection in a mouse model, showed a 50% decrease in retinal neovascularization in the deep plexus. Data indicate that the anti‐miRNA exerts its antiangiogenic effects by acting on ECs and through Vascular endothelial growth factor (VEGF) and Basic fibroblast growth factor (bFGF) pathways 71, 99. Moreover, following the initiation of malignancy, there is poor vascularization of the tumour mass which leads to stressful conditions in the tumour microenvironment, including hypoxia, nutrient deprivation and pH changes. In such circumstances, down‐regulation of VEGF and bFGF pathways by miRNAs may provide a means by which tumour cells cannot escape cell death initiation 96, 99.

Data indicate that EC‐specific miRNAs are potentially interesting therapeutic targets that can be used to inhibit the initiation of the ischaemic‐induced pathological processes.

According to evidence, miRNAs act as critical regulators of EC survival and pathophysiology of angiogenesis. For instance, in patients with PAD, mRNAs of P‐selectin glycoprotein ligand (*SELPG*), *ICAM1*,* ITGA6*, (*F11R*), and NF‐κB are all up‐regulated targets of PAD‐specific miRNAs. SELPG binds to E‐, L‐, and P‐selectins 7, 45. The finding that under ischaemic complications, up‐regulation of miRNAs such as miR‐15s, miR‐23s and miR‐503 contributes to ischaemic‐induced impairment of EC function and reparative angiogenesis considerably advances our understanding on the mechanisms used by miRNAs to control angiogenesis 29, 45, 80, 83.

In this context, antagonism of endogenous miRNA regulators such as miR‐15s, miR‐23s and miR‐222s that are induced under pathological conditions and also target ‘gatekeepers’ of endothelial activation may represent a powerful strategy to inhibit or activate angiogenesis in a wide range of pathological conditions 29, 45, 71.

In contrary, a distinct set of miRNAs including miR‐17‐92, ‐126, ‐130a, ‐133 has been associated with compensatory arteriogenesis to regulate vascular development 8, 10, 24, 86, 87.

The miR‐17–92 cluster has been implicated not only in ischaemic‐induced complications and cellular senescence but also in cell proliferation and tumour growth. Increased expression of miR‐17‐92 cluster is usually associated with oncogenesis, cell growth and proliferation, while decreased expression of the miR‐17–92 cluster are generally associated with cellular senescence 15, 24, 96.

Data also indicate that specific miRNAs such as miR‐130, ‐132, ‐133 are potentially interesting therapeutic targets that can be used to inhibit the initiation of the ischaemic‐induced pathological processes effects by acting on ECs and through VEGF and bFGF pathways 71, 86, 87, 99.

**Figure 3:**
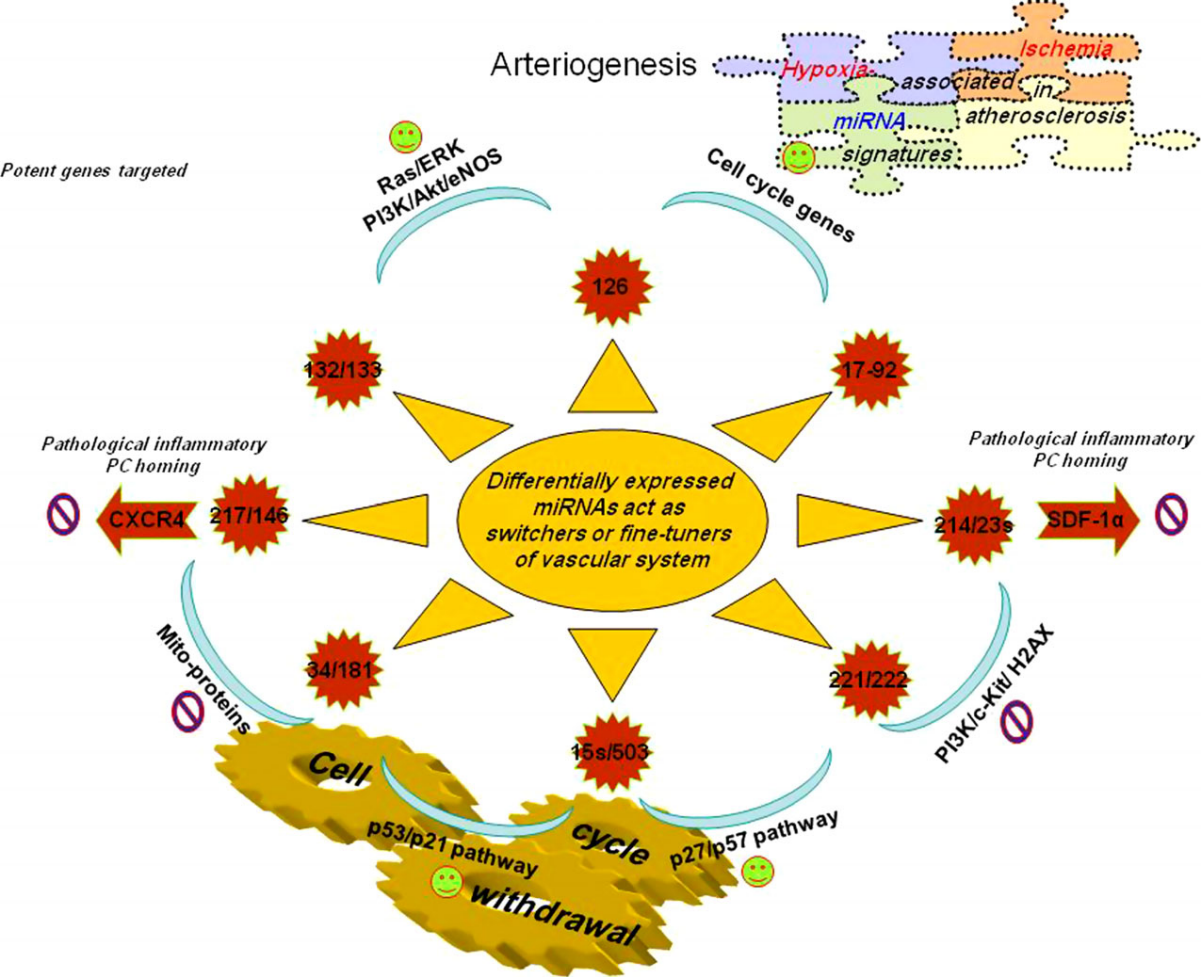
Diagrammatic illustration of potentially miRNAs involved in angiogenesis related changes (good or bad).

Conclusively, combination of miRNA‐based strategies will potentially refine cell therapies while increasing therapeutic administration routes and effectiveness of treatment, while neutralization of ischaemic‐induced miRNAs with antagomirs would suppress pathological conditions associated with ischaemia, while pointing towards a new therapeutic strategy for diseases associated with retinal and tumour angiogenesis 80, 83 (Fig. 3).

## Circulating miRNAs associated with myocardium function

As humans age, the functions of vascular system deteriorates, resulting in the development of ischaemia‐related degenerative disease. miRNAs released from adult degenerating organ affected by ageing, control stem cell renewal of various organs including EC function of vascular system and heart.

It is well known that circulating miRNAs are taken up into other cells or organs for the induction of specific functions. Circulating levels of miRNAs predict the regression potential of ischaemic‐related disorders in patients with EC dysfunction.

Several human studies showed that the proliferation, differentiation and migration capacity of EPCs and tissue‐specific PCs are regulated by circulating miRNAs. Thus, EC dysfunction and ageing‐related degenerative problems lie, at least in part, within the tissue‐specific released miRNAs 6, 15, 29, 50, 51, 80.

The exosomes and micro‐particles enriched in miRNAs are released from the cells under stress and are subsequently internalized by the other cells. For example, after MI circulating miR‐1 and ‐133a released from exosomes can be taken up into other cells or organs for the induction of specific functions 87, 88, 89.

One of instances is elevated levels of circulating miR‐1 and ‐133a in patients with CVD originate mainly from the injured myocardium and can induce the function of other organs as a signalling marker for cardiomyocyte death.

While circulating levels of miR‐1 and ‐133 are elevated, in patients with ischaemic cardiomyopathy or after MI, however, the expression levels of miR‐1 and ‐133 are diminished in diseased human heart 70, 82, 87, 90.

*In vivo* administration of miR‐1 and ‐133 in animal models protects against adverse effects after MI induced by chronic ischaemia 88, 89, 91 and causes reprogramming of foetal‐associated cardiac genes including, atrial natriuretic factor and skeletal muscle α‐actin (Actα1) 82, 90. Accordingly, miR‐1 and ‐133 simultaneous administration is affecting multiple processes associated with ischaemia‐inducing pathological conditions, and can prevent from heart failure 93, 94. Intriguingly, administration of miR‐133a and epigenetic factor miR‐29 would inhibit cardiac fibrosis and apoptosis, by down‐regulating voltage‐gated outward K (Kv) channels 92.

Consistent with these findings, data indicate that in mouse and monkeys, the levels of miR‐1 and miR‐133 are inhibiting histone deacetylases and increasing acetylation of histones and GATA4 96, 97. GATA4 and p300 form a transcriptional complex, to induce the expression of specific genes in tissues receive them.

**Figure 4:**
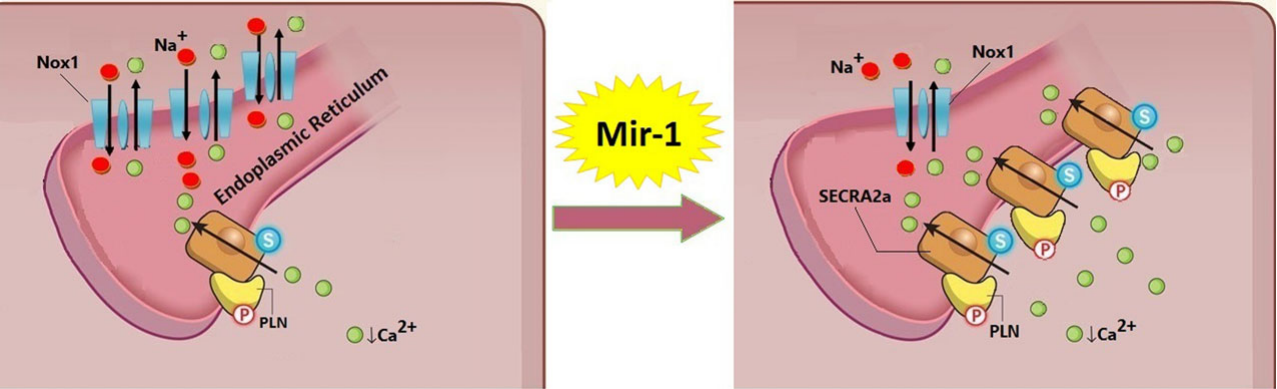
A proposed model for circulating levels of miRNAs predicts the regression potential of ischaemic‐related disorders in patients with EC dysfunction. The potential role of miR‐1 regulates intracellular Ca^2+^ homoeostasis to suppress progression towards the intracellular Ca^2+^ signalling impairment. The miR‐1 up‐regulates some of genes that are down‐regulated in ischaemic failing heart. miR‐1 markedly down‐regulates the hypertrophic‐associated genes α‐MHC and Ncx1, while strongly up‐regulates failing‐suppressing ATPase pomp Serca2a for efficient Ca^2+^ uptake into SR. α‐MHC: Cardiac alpha‐myosin heavy chain; Ncx1: Sarcolemmal sodium/calcium exchanger; Serca2a: Cardiac SR Ca^2+^‐ATPase; SR: Sarcoplasmic reticulum.

In addition, miR‐1 plays potential role in intracellular Ca^2+^ homeostasis. The miR‐1 causes a strong up‐regulation of Serca2a expression, indicative of enhanced SR Ca^2+^ cycling, which correlates with the subsequent improvement in myocyte contractility (Fig. 4). The Ca^2+^ uptake into SR is mediated by SR Ca^2+^‐ATPase (Serca2a) and regulated by Pln. Serca2a is an important regulator of intracellular Ca^2+^ signalling 82.

There is also reports that elevated levels of miR‐1 target the anti‐apoptotic factor IGF‐1 in induced stem cells. IGF‐1 regulates proliferation and survival of various cell types through regulation of multiple pathways including mitochondrial cytochrome‐c/caspase pathway 80. In response to miR‐1, the phosphorylation levels of both ERK1/2 and p38 kinases are increased whereby enhance their activities. Also, miR‐1 causes is up‐regulation of anti‐apoptotic gene Bcl‐2 *versus* pro‐apoptotic gene Bax 98.

## Conclusion

Ischemic complications represent leading cause of morbidity and mortality in atherosclerotic patients and contribute to age‐related diseases. Atherosclerosis is the primary cause of CVDs. The overall impact on heart is tissue hypoperfusion, myocardium infarction and heart failure. Moreover, the cell death in ischemic conditions and inappropriate angiogenesis worsen the recovery from an ischemic insult. In ischemic complications, appropriate neo‐vascularization is a potential therapeutic target for improving healing from ischemic injuries. Proper molecular stimulation of angiogenesis process represents a novel strategy to support post‐ischemic blood flow recovery, protection from wound closures, and tissue regeneration. This study tries to show that miRNAs are differently expressed in patients with ischemic complications. Furthermore, miRNAs act as critical regulators of survival and migration of PCs to improve reparative angiogenesis.

The objective of current study according to preliminary evidence, was (*i*) describing the critical roles of miRNAs in cell death and angiogenesis during ischemic conditions; (*ii*) clarifying putative roles of miRNAs to protect against ischemia complications; and finally (*iii*) declaring them as targets in ischemic preconditioning protection responses.

## Conflicts of interest

The authors confirm that there are no conflicts of interest.
